# Supplementary material for: Evaluation of Immunogenicity of an Orf Virus Vector-Based Vaccine Delivery Platform in Sheep
Source: Vaccines (Basel). 2025 Jun 11;13(6):631. doi: 10.3390/vaccines13060631 (PMC12197756; doi:10.3390/vaccines13060631)
Supplement: Supplementary file 1 [file vaccines-13-00631-s001.zip › Supplementary Figure S1.pdf]

vaccines

MDPI

Type of the Paper (Article)

(A)

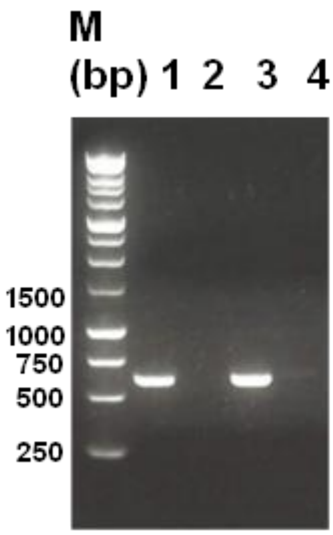

(B)

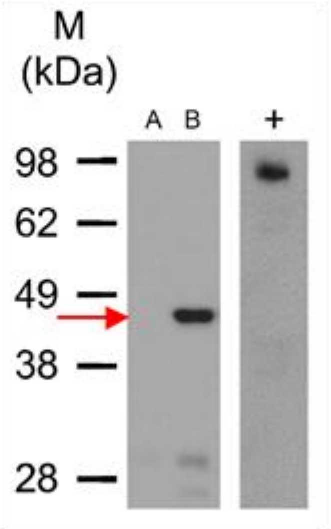

Figure S1. *OmpA* (mRNA) and OmpA-Flag protein expression of *ompA*-based vaccines *in vitro*. (A) *OmpA* RT-PCR for FLS infected with mORFV-*ompA*. FLS cells were inoculated with mORFV-*ompA* for 3 and 6 days post infection and harvested and analysed as described in section 2.2. An agarose gel of the *ompA* RT-PCR (product size 594bp) is displayed in a 1% agarose gel in TAE buffer and GelRed. The lanes display: M- marker, 1Kb ladder (Promega), 1, mORFV-*ompA* day 3 RT + reaction; 2, mORFV-*ompA* day 3 RT- reaction, 3, mORFV-*ompA* day 6 RT + reaction; 4, mORFV-*ompA* day 6 RT- reaction.

(B) Western blot for MOMP of CRFK cells transduced with MVV-*ompA*. CRFK cells were transduced with MVV vectors encoding either *EGFP* or *ompA* and 72 hours later cell lysates proteins were visualised by immunoblotting using a monoclonal antibody to the C-terminal FLAG epitope. A. MVV-EGFP; B. MVV-ompA. Red arrow indicates the expected size of MOMP-FLAG. Positive control (+) is an unrelated FLAG-tagged protein as technical control.
