# Supplementary material for: Evaluation of Immunogenicity of an Orf Virus Vector-Based Vaccine Delivery Platform in Sheep
Source: Vaccines (Basel). 2025 Jun 11;13(6):631. doi: 10.3390/vaccines13060631 (PMC12197756; doi:10.3390/vaccines13060631)
Supplement: Supplementary file 1 [file vaccines-13-00631-s001.zip › Supplementary Table S1.pdf]

vaccines

MDPI

Type of the Paper (Article)

Group	Animal	Baseline (bleed 2 day -18)	Post-primary immunisation (bleed 3, day 10)	Post-booster immunisation (bleed 6, day 49)
**mMVV-*ompA***	**2**	21.6	15.49	42.71
**mMVV-*ompA***	**16**	11.62	8.47	7.86
**mMVV-*ompA***	**26**	10.16	10.28	14.4
**mMVV-*ompA***	**27**	10.95	7.14	57.11
**ORFVV-*ompA***	**1**	8.59	12.16	157.96
**ORFVV-*ompA***	**3**	6.35	6.47	136.54
**ORFVV-*ompA***	**9**	9.01	10.83	9.13
**ORFVV-*ompA***	**20**	9.86	8.35	248.76
**Unvaccinated control**	**8**	34.61	27.08	23.45
**Unvaccinated control**	**10**	16.48	24.14	17.29
**Unvaccinated control**	**28**	23.74	20.36	14.72
**Unvaccinated control**	**30**	72.38	32.91	37.13
***C. abortus* positive**	**31**	496.32	498.58	439.00

Table S1. Study MOMP antibody data for three bleeds: Pre-immunisation (bleed 1), post primary immunisation (bleed 3) and post booster immunisation (bleed 6). Serum samples were tested using an ID Screen® *Chlamydophila abortus* Indirect Multi-species ELISA kit MOMP ELISA (Section 2.8). The data from the *C. abortus* positive is serum tested from a post-abortion convalescent sheep.
