# Supplementary figures and images for: Evaluation of Immunogenicity of an Orf Virus Vector-Based Vaccine Delivery Platform in Sheep
Source: Vaccines (Basel). 2025 Jun 11;13(6):631. doi: 10.3390/vaccines13060631 (PMC12197756; doi:10.3390/vaccines13060631)

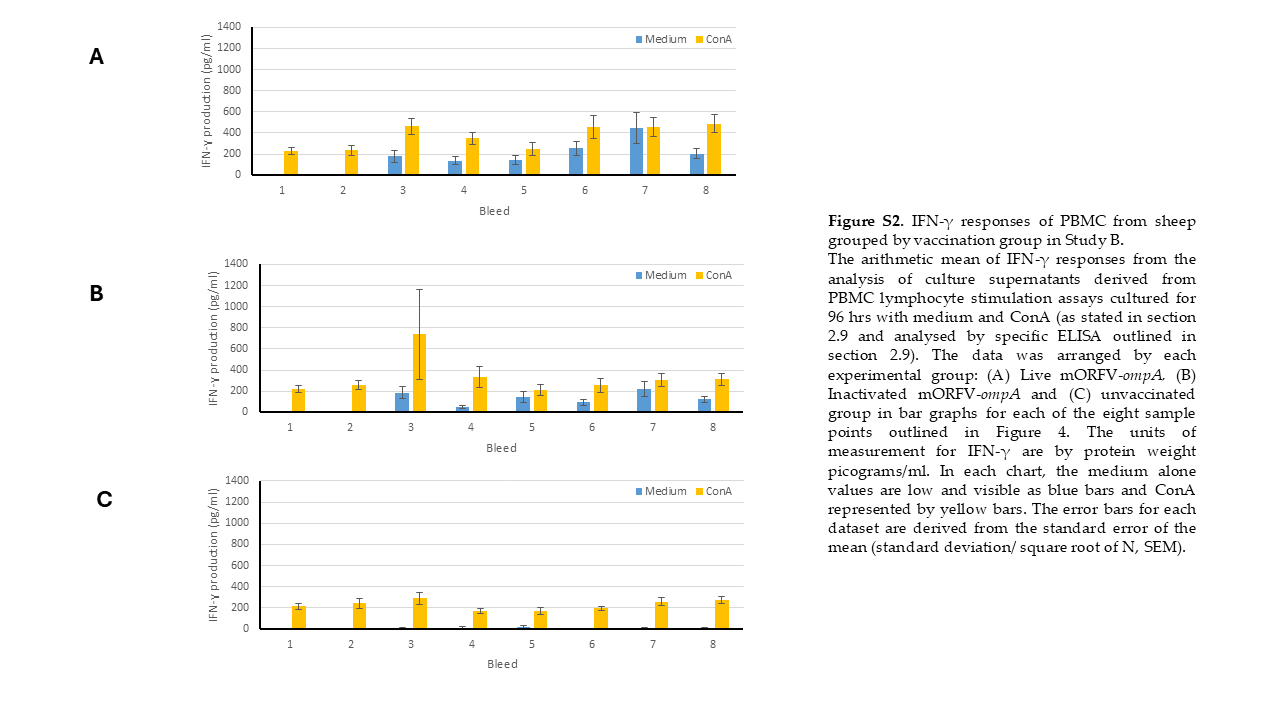

Supplement: Supplementary file 1 [file vaccines-13-00631-s001.zip › Supplementary Figure S2 IFNg.tif]

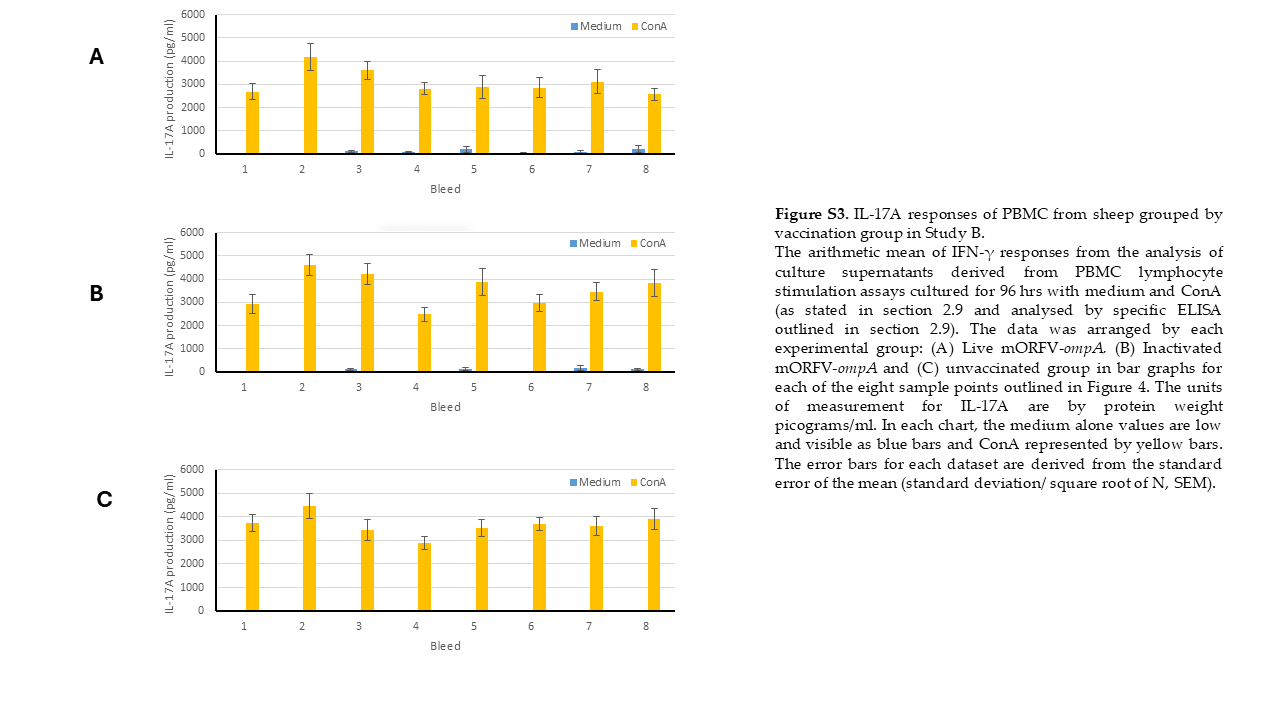

Supplement: Supplementary file 1 [file vaccines-13-00631-s001.zip › Supplementary Figure S3 IL-17A.tif]

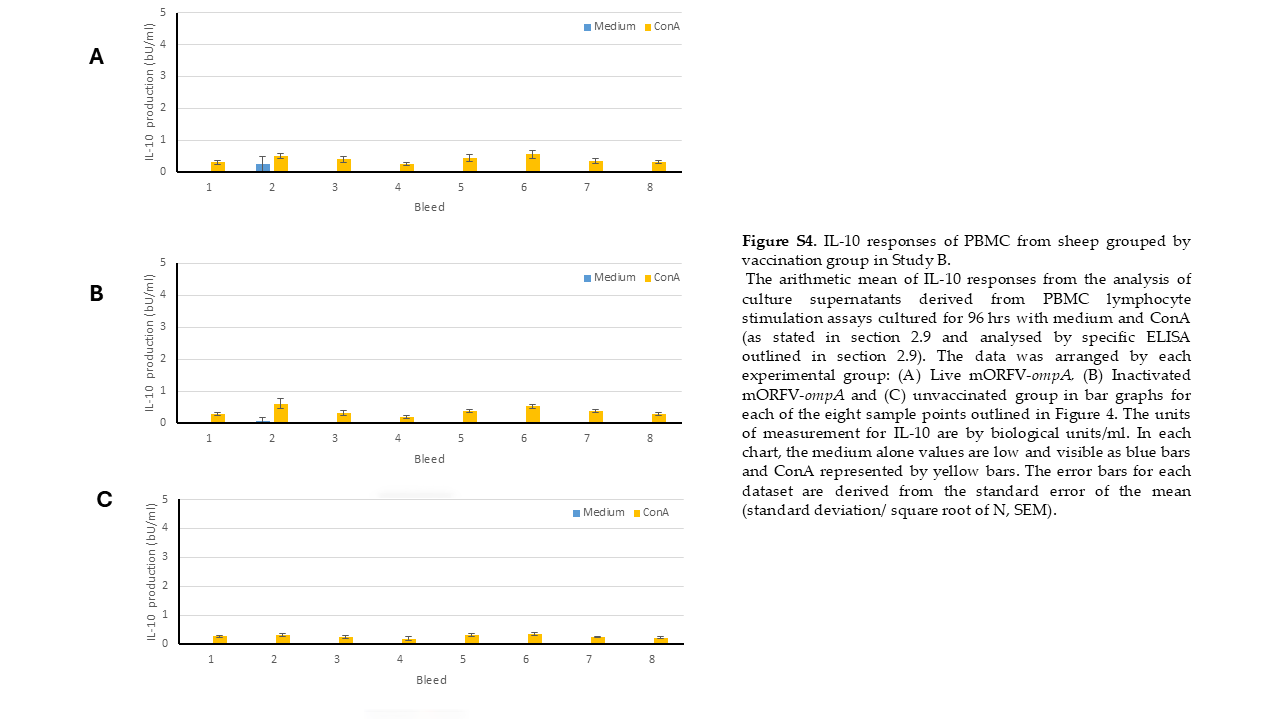

Supplement: Supplementary file 1 [file vaccines-13-00631-s001.zip › Supplementary Figure S4 IL-10.tif]

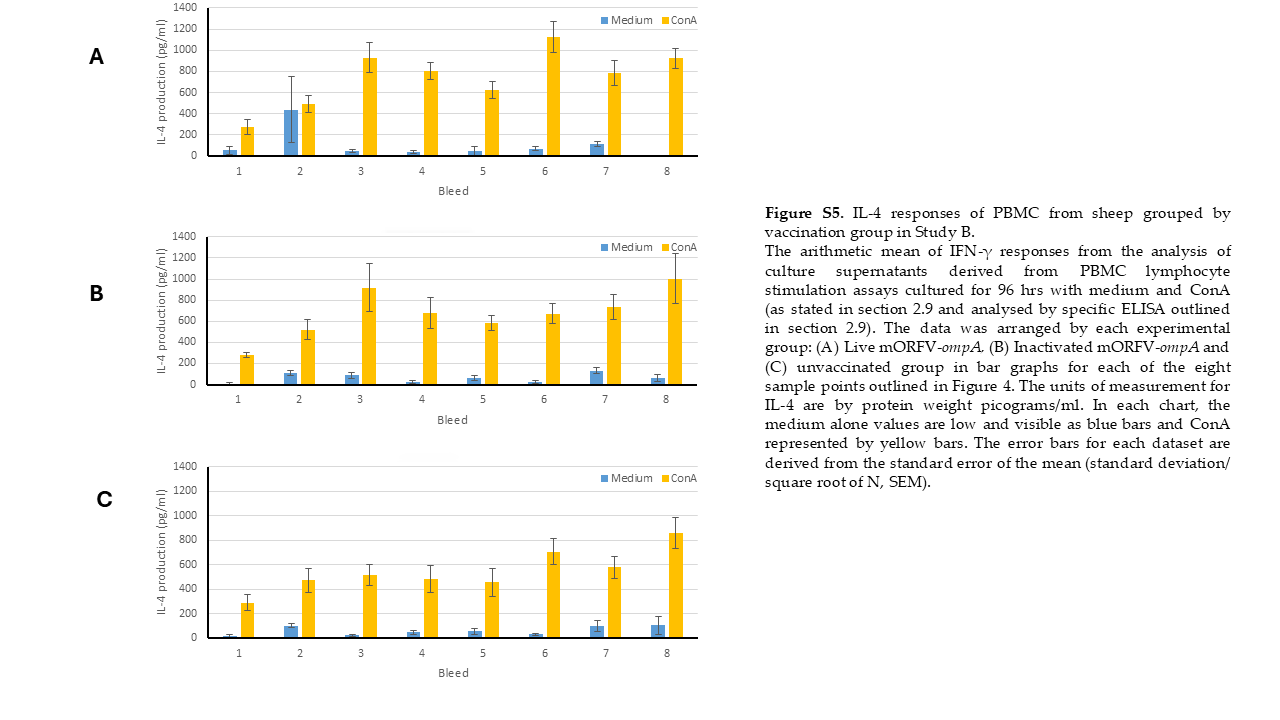

Supplement: Supplementary file 1 [file vaccines-13-00631-s001.zip › Supplementary Figure S5 IL-4.tif]
